# Plasma Calprotectin Is Predictive for Short-Term Functional Outcomes of Acute Ischemic Stroke

**DOI:** 10.3389/fneur.2022.811062

**Published:** 2022-03-21

**Authors:** Zicheng Hu, Haihua Li, Yongping Zhu, Jun Zhang, Xiao Yang, Rongzhong Huang, Yongyong Li, Haitao Ran, Tingting Shang

**Affiliations:** ^1^Department of Neurology, People's Hospital of Chongqing Hechuan (PHHC), Chongqing, China; ^2^Neuroscience Center, General Hospital of Ningxia Medical University, Yinchuan, China; ^3^Department of Gerontology, The Second Affiliated Hospital of Chongqing Medical University, Chongqing, China; ^4^Department of Ultrasound, The Second Affiliated Hospital of Chongqing Medical University, Chongqing, China

**Keywords:** acute ischemic stroke, biomarker, calprotectin, functional outcome, inflammation

## Abstract

**Background:**

Blood-based prognostic biomarkers of acute ischemic stroke (AIS) are limiting. Calprotectin is suggested to be involved in directing post-stroke inflammatory conditions. However, the pathological alteration of circulating calprotectin in AIS is yet to be thoroughly elucidated. Therefore, this study aimed to investigate the levels and clinical relevance of calprotectin in AIS.

**Methods:**

This study recruited 271 patients with AIS within 24 h since symptom onset and 145 non-stroke healthy controls (HC) from February 1, 2018, to Dec 31, 2020. Patients were followed up for 2 weeks for observation of functional outcomes, as determined by the National Institutes of Health Stroke Scale (NIHSS) and modified Rankin Scale (mRS). Plasma calprotectin concentrations were determined by ELISA.

**Results:**

Plasma calprotectin concentrations were significantly higher in patients with AIS compared with controls [patients vs. control: median (IQR) 54.2 (39.01–99.04) vs. 50.04 (35.42–61.22), *p* < 0.001]. Besides, patients with poor prognosis, as defined by mRS ≥ 3, had significantly higher calprotectin levels than patients with good prognosis [poor prognosis patients vs. good prognosis patients: median (IQR) 61.99 (47.52–108) vs. 43.36 (33.39–60.2), *p* < 0.001]. Plasma calprotectin levels were positively associated with the disease severity of AIS, as reflected by infarction volume and NIHSS score at baseline. Furthermore, baseline calprotectin was found to be independently associated with poor prognosis [odds ratio (OR): 1.02, 95% CI: 1.01–1.03] and disease progression (OR: 1.03, 95% CI: 1.02–1.04) of AIS during a 2-week follow-up, with adjustment of possible confounding factors.

**Conclusion:**

Plasma calprotectin is associated with short-term functional outcomes of AIS.

## Introduction

Acute ischemic stroke (AIS) is one of the leading causes of mortality and disability worldwide (1). Over the next years, the global AIS burden is expected to increase steadily because of population aging. The pathophysiology of brain ischemia and post-ischemia changes in the brain are not yet fully understood. Inflammation is increasingly recognized as an important element in the pathogenesis of ischemic stroke. Brain ischemia leads to an immediate local inflammatory reaction with activation of microglia, astrocytes, and endothelial cells, as well as the further release of pro-inflammatory cytokines (2). These non-specific alterations post-ischemia could result in blood-brain barrier dysfunction and infiltration of peripheral inflammatory cells and cytokines into the brain, which may further increase tissue injury (3).

A panel of inflammatory molecules are predictive markers of severity and functional outcomes of AIS; such molecules are proposed as potential therapeutic targets in AIS (4). Calprotectin, which is formed by S100A8 and S100A9 heterodimer, plays a pivotal role in promoting inflammation and atherosclerosis progression. Plasma calprotectin is associated with the risk of coronary artery events (5) and peripheral artery diseases (6). Moreover, circulating calprotectin has been demonstrated to be elevated in AIS (7), and inhibition of S100A9 has been found to suppress thrombus formation in experimental models of AIS (8). However, there is limited evidence currently about the predictive effects of calprotectin on short-term functional outcomes of AIS. Therefore, this study aimed to investigate the potential of circulating calprotectin as a short-term prognostic biomarker of AIS.

## Materials and Methods

### Subjects

**Figure 1:**
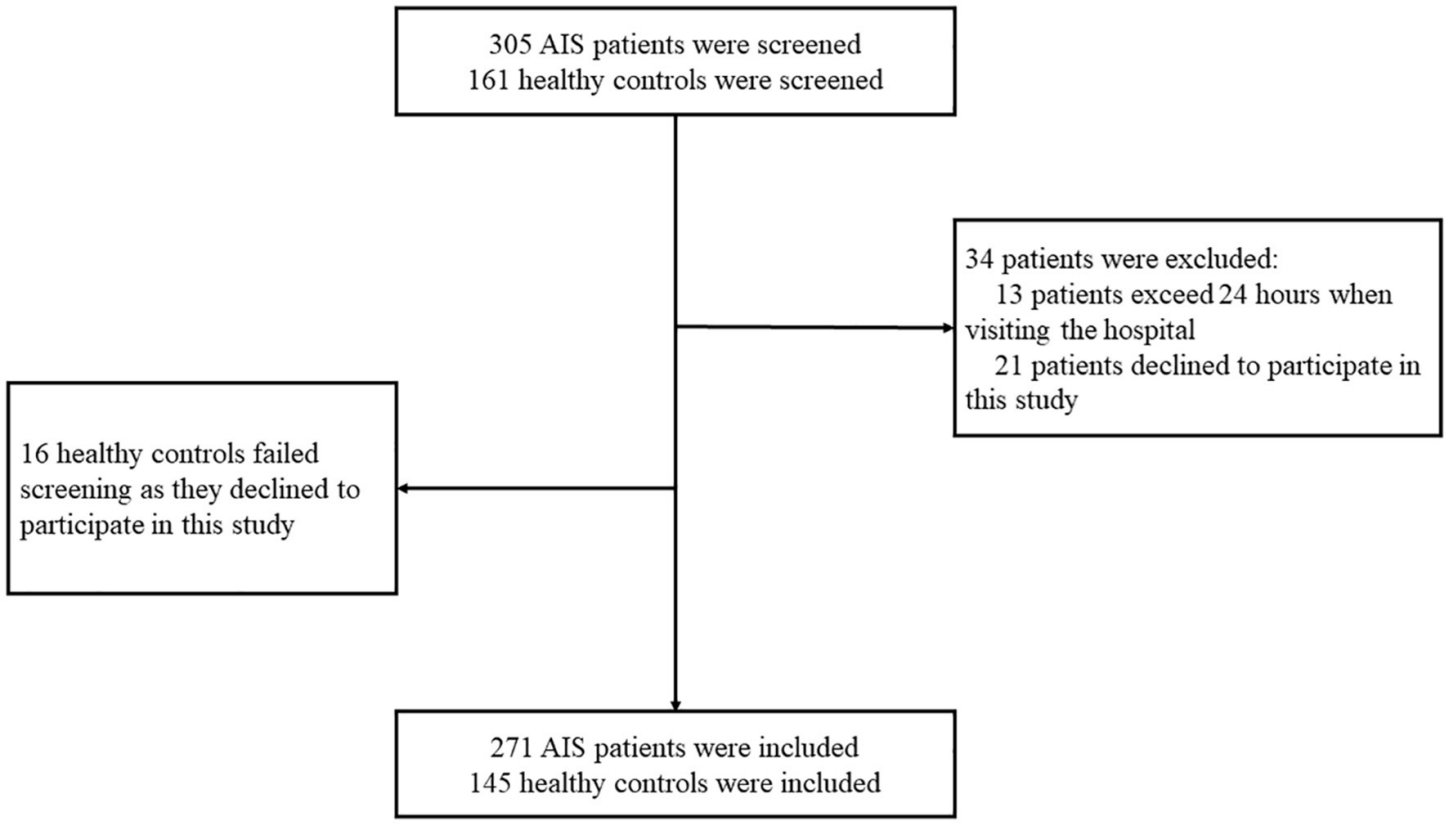
Participants screening flowchart. In this study, 305 patients with acute ischemic stroke (AIS) and 161 non-stroke controls were screened for eligibility for participation. A total of 34 patients with AIS failed screening for the following reasons: (1) 13 patients visited the hospital beyond 24 h since symptom onset; (2) 21 patients declined to participate. Finally, 16 non-stroke controls failed the screening since they declined to participate.

In this study, 305 patients with first-ever AIS who were hospitalized at the Department of Neurology, the Second Affiliated Hospital of Chongqing Medical University, during February 1, 2018 and July 31, 2021 were screened for eligibility for participation. The inclusion criteria included the following: (1) newly onset AIS; (2) visited the hospital within 24 h after symptom onset; (3) willingness to participate. A total of 161 age- and sex-matched non-stroke subjects were screened for eligibility for participation in the health examination center of this hospital. Subjects were excluded if they have one of the following conditions: (1) declined to participate; (2) any type of tumors; (3) exceeds 24 h when visiting the hospital. Finally, 271 patients and 145 age and sex-matched non-stroke controls were recruited in this study (Figure 1). Written informed consents for participation and blood sampling were obtained from all participants or their legal relatives. This study conformed with the principles of the Declaration of Helsinki and was approved by the investigational review board of the Second Affiliated Hospital of Chongqing Medical University and the People's Hospital of Chongqing Hechuan (Approval No. L2017HCDH0912).

### Clinical Evaluation

At baseline, the demographic information of the patients, including age, sex, and body mass index (BMI), was collected and assessed upon admission. The following pieces of information were also obtained: pre-stroke medical history including the use of oral anticoagulants (such as Warfarin and novel oral anticoagulants, e.g., Dabigatran and Rivaroxaban) or antiplatelet drug (such as Clopidogrel, Aspirin, and Ticagrelor), and comorbidities, including hypertension, diabetes mellitus, hypercholesteremia, and atrial fibrillation. AIS was diagnosed according to the WHO Multinational Monitoring of Trends and Determinants in Cardiovascular Disease (WHO-MONICA) criteria and was verified by MRI reports, which were performed within 24 h after patient admission. The neurological deficits were examined using the National Institutes of Health Stroke Scale (NIHSS) upon admission (9), which was performed by a certified stroke neurologist. The stroke subtype was determined with the TOAST criteria.

### Patients' Follow-Up

Patients were followed up for 2 weeks since admission for the observation of neurological functions. The primary endpoint was functional outcomes which were observed 2 weeks since symptom onset. Functional outcomes were assessed by NIHSS and the modified Rankin Scale (mRS) (10), which were blinded to plasma calprotectin.

### Blood Sampling and Measurement of Calprotectin

Venous blood samples were drawn from all patients immediately after patients' visiting before the initiation of any treatment. These blood samples were centrifuged at 1,200 × *g* for 10 min, 4°C immediately after collection, and stored at −80°C until use. Citrated plasma samples were thawed on ice and thoroughly vortexed before the test of calprotectin concentrations (Abcam, USA). All samples were tested in duplicates and the means of each measurement were used for analysis. For samples with extreme numbers which distribute beyond means ± 3 *SD*, the analysis would be repeated with three previously validated samples as references. These experiments were performed in a blinded manner following the manufacturer's instructions.

### Statistical Analysis

Continuous variables were tested for normality; if normally distributed, an independent *t*-test was used to determine the difference between groups. If continuous variables were not normally distributed, a Mann-Whitney *U*-test was used. Two-sample tests of proportions (for categorical data) were used to compare proportions. Spearman correlation analyses were utilized to investigate associations between calprotectin and other factors. The receiver operating characteristic (ROC) curve analyses were utilized to test the predictive effects of baseline parameters on short-term functional outcomes. Optimal sensitivity and specificity were determined *via* a non-parametric approach. The Youden index was calculated for the cutoff value to determine the cutoff value that maximized the discriminating power of the test. Multivariate adjusted logistic regression models were utilized to assess the association between plasma calprotectin levels and functional outcomes. The first logistic regression model included the “poor prognosis” (as indicated mRS ≥ 3 or death) as the dependent variable. The second logistic regression model included “disease progression” as the dependent variable. “Disease progression” was defined as either an increase of NIHSS score or death during follow-up. Specifically, if a patient's NIHSS score at the endpoint was higher than that at baseline or the patient died during follow-up, this patient would be allocated into the “disease progression” group. Age, sex, BMI, hypertension, diabetes mellitus, hyperlipidemia, atrial fibrillation, smoking, family history of stroke, antiplatelet, and anticoagulation drug use were adjusted when investigating the association between these factors and outcomes. When analyzing the association of NIHSS at admission, infarction volume, time from symptom onset, recombinant tissue plasminogen activator (rtPA) treatment, and calprotectin with functional outcomes, all aforementioned factors were adjusted. A *p*-value ≤ 0.05 was recognized as statistically significant. Statistical analyses were conducted using SPSS statistical package version 24 (IBM SPSS Statistics for Windows, Armonk, NY, USA).

## Results

### Demographic Characteristics of Subjects

This study included 271 patients with AIS and 145 non-stroke controls. Patients and controls had no significant difference in the mean age, frequencies of men, subjects with a smoking history, subjects with a history of antiplatelet and anticoagulation drug use, and those with a family history of stroke. However, patients with AIS had a significantly higher median BMI than controls. Patients with AIS had significantly higher frequencies of hypertension and diabetes mellitus than those in the control group. No difference was found in the frequencies of hypercholesteremia and atrial fibrillation between the two groups. Further results showed that 150 (55.35%) patients with AIS have atherothrombotic type stroke, 15 (5.54%) patients have cardioembolic type stroke, 95 (35.06%) patients have lacunar type stroke, and the rest have unknown type stroke. On later findings, 9 (3.32%) patients with AIS who were recruited in this study had died, 7 (2.58%) had hemorrhagic transformations, 44 (16.24%) patients had disease progression as indicated by an increase of NIHSS score during follow-up, and 160 (59.04%) patients had a mRS ≥ 3 at endpoint (Table 1).

**Table 1:** Demographic data of subjects.

	**Patients (*n* = 271)**	**Controls (*n* = 145)**	***P*-value**
Age, year (SD)	65.72 (8.84)	65.88 (10.69)	0.876^a^
Male, No. (%)	128 (47.23)	69 (47.59)	1.000^b^
BMI, Median (IQR)	25.05 (23.63, 26.21)	23.86 (22.73, 25.81)	**<0.001^c^**
Smoking history, No. (%)	49 (18.08)	28 (19.31)	0.792^b^
Antiplatelet drug use, No. (%)	34 (12.55)	19 (13.10)	0.878^b^
Anticoagulation drug use, No. (%)	7 (2.58)	5 (3.45)	0.760^b^
Family history of stroke, No. (%)	22 (8.12)	10 (6.90)	0.704^b^
**Comorbidities**			
Hypertension, No. (%)	131 (48.34)	44 (30.34)	**<0.001^b^**
Diabetes Mellitus, No. (%)	77 (28.41)	21 (14.48)	**0.002^b^**
Hypercholesteremia, No. (%)	39 (14.39)	28 (19.31)	0.209^b^
Atrial Fibrillation, No. (%)	16 (5.90)	6 (4.14)	0.500^b^
DWI volume, ml, Median (IQR)	34 (15, 53)	NA	NA
NIHSS at admission, Median (IQR)	10 (6, 16)	NA	NA
NIHSS at 2 weeks, Median (IQR)	10 (5, 12)	NA	NA
mRS at 2 weeks, Median (IQR)	3 (1, 4)	NA	NA
**Stroke etiology**			
Large artery atherothrombotic, No. (%)	150 (55.35)	NA	NA
Cardioembolic, No. (%)	15 (5.54)	NA	NA
Small artery occlusion, No. (%)	95 (35.06)	NA	NA
Others, No. (%)	11 (4.06)	NA	NA
**Endpoint event**			
Death, No. (%)	9 (3.32)	NA	NA
Hemorrhagic transformation, No. (%)	7 (2.58)	NA	NA
Disease progression, No. (%)	44 (16.24)	NA	NA
mRS ≥ 3, No. (%)	160 (59.04)	NA	NA

*IQR, Inter-Quartile Range; BMI, Body Mass Index; DWI, diffusion-weighted imaging; NIHSS, National Institutes of Health Stroke Scale*.

a *Unpaired t-test*.

b *Pearson χ^2^-test*.

c *Mann-Whitney U-test*.

*If the patient's NIHSS at the endpoint was higher than that at baseline or the patient died during follow-up, it was defined as “disease progression.” “Poor prognosis” was defined as mRS ≥ 3 or death. P values less than 0.05 were boldfaced*.

### Plasma Levels and Clinical Relevance of Calprotectin in Patients With AIS

**Figure 2:**
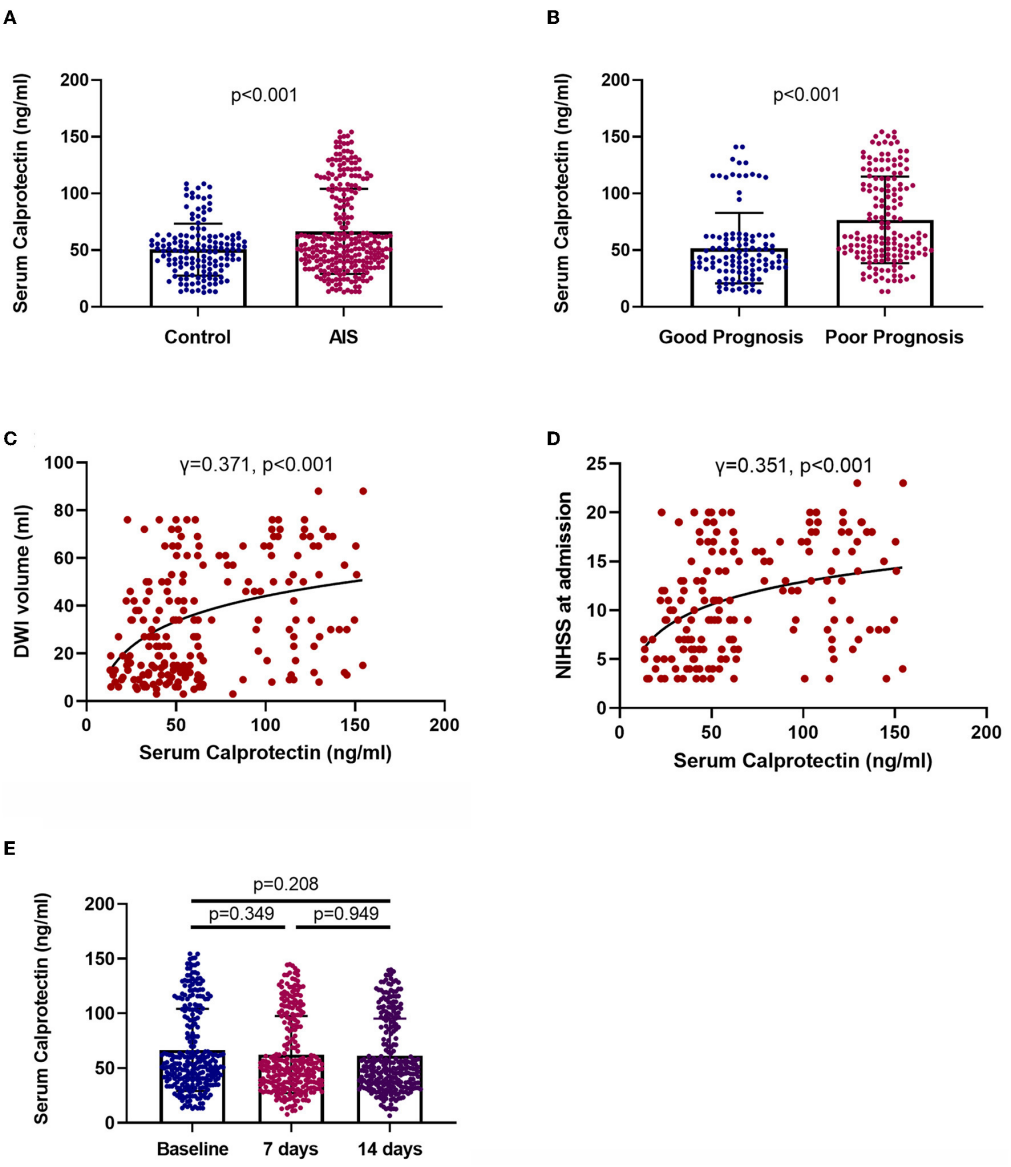
Levels and clinical relevance of plasma calprotectin in AIS. **(A)** Plasma calprotectin levels in patients with AIS and controls. Unpaired *t*-test. **(B)** Plasma calprotectin levels in patients with AIS with good and poor prognosis. Poor outcome is defined as mRS ≥ 3 or death. Unpaired *t*-test. **(C)** Association between plasma calprotectin levels and diffusion-weighted imaging (DWI) hyperintensity volume at baseline. Spearman correlation analysis. **(D)** Association between plasma calprotectin levels and National Institutes of Health Stroke Scale (NIHSS) scores at baseline. Spearman correlation analysis. **(E)** Dynamic change of serum calprotectin during the 2-week follow-up after AIS. One-way ANOVA.

In comparison with controls, patients with AIS had significantly higher plasma levels of calprotectin [patients vs. control: median (IQR) 54.2 (39.01–99.04) vs. 50.04 (35.42–61.22), *p* < 0.001] (Figure 2A). Moreover, patients with AIS along with poor prognosis had significantly higher plasma levels of calprotectin than patients with good prognosis [poor prognosis patients vs. good prognosis patients: median (IQR) 61.99 (47.52–108) vs. 43.36 (33.39–60.2), *p* < 0.001] (Figure 2B). Plasma calprotectin concentrations were positively correlated with diffusion-weighted imaging (DWI) hyperintensity volume (γ = 0.371, *p* < 0.001, Figure 2C) and NIHSS scores at baseline (γ = 0.351, *p* < 0.001, Figure 2D). These findings indicated that plasma calprotectin levels were increased in patients with AIS and were associated with the disease severity of AIS, as reflected by infarction volume and NIHSS scores. Notably, the NIHSS score at baseline was significantly correlated with DWI hyperintensity volume in patients with AIS. Furthermore, we found in this study that serum calprotectin concentrations had no significant change during a 2-week follow-up (Figure 2E), indicating that circulating calprotectin levels are relatively stable after AIS.

### Predictive Effects of Baseline Plasma Calprotectin on Short-Term Functional Outcomes of AIS

**Figure 3:**
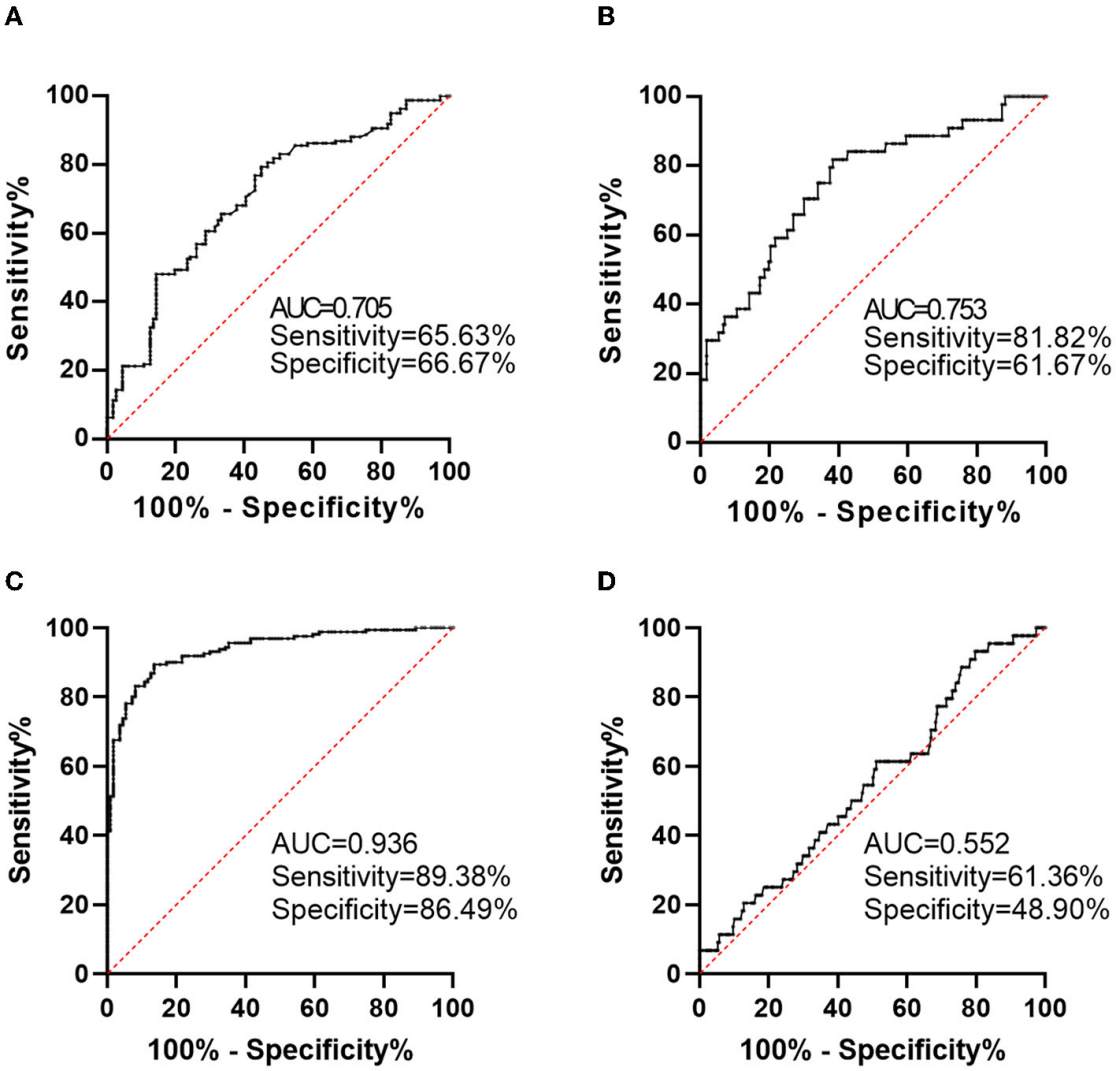
Predictive value of calprotectin for short-term functional outcomes of AIS. **(A)** Predictive value of calprotectin for poor outcome 2 weeks after AIS onset as reflected by mRS ≥ 3. **(B)** Predictive value of calprotectin for disease progression during a 2-week follow-up as reflected by an increase in NIHSS score. **(C)** Predictive value of the combination of calprotectin, infarction volume, and NIHSS at admission for poor outcome 2 weeks after AIS onset. **(D)** Predictive value of the combination of calprotectin, infarction volume and NIHSS at admission for disease progression during a 2-week follow-up.

We conducted ROC analyses to investigate the predictive effects of baseline plasma on short-term outcomes of AIS. We primarily conducted this analysis to explore the predictive effects of calprotectin for poor prognosis as reflected by mRS ≥ 3 or death during follow-up. We found that baseline calprotectin has a relatively high accuracy in predicting poor prognosis, which generated a sensitivity of 65.63%, a specificity of 66.67%, and an area under the ROC curve (AUC) of 0.705 (Figure 3A). The predictive effects of calprotectin for disease progression generated a sensitivity of 81.82%, a specificity of 61.67%, and an AUC of 0.753 (Figure 3B). The combination of calprotectin, infarction volume, and NIHSS at admission predicted a poor prognosis of AIS with a sensitivity of 89.38%, a specificity of 86.49, and an AUC of 0.936 (Figure 3C). The combination of calprotectin, infarction volume, and NIHSS at admission predicted disease progression of AIS with a sensitivity of 61.36%, a specificity of 48.9, and an AUC of 0.552 (Figure 3D).

### Risk Factors of Poor Functional Outcome and Disease Progression in AIS

**Figure 4:**
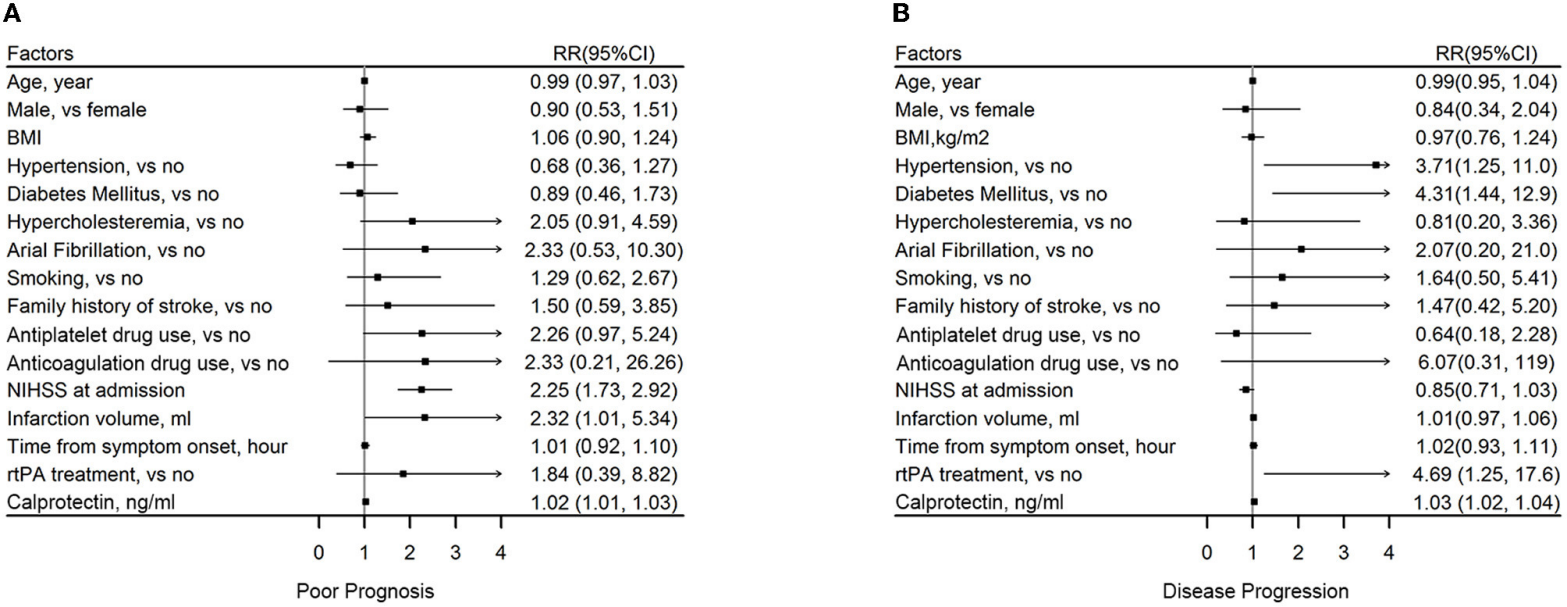
Association between plasma calprotectin and short-term functional outcomes of AIS. **(A)** Risk factors of poor outcome 2 weeks after AIS onset as reflected by mRS ≥ 3. **(B)** Risk factors of disease progression during a 2-week follow-up as reflected by an increase in NIHSS score.

National Institutes of Health Stroke Scale at admission [odds ratio (OR): 2.25, 95% CI: 1.73–2.92], infarction volume (OR: 2.32, 95% CI: 1.01–5.34), and baseline calprotectin (OR: 1.02, 95% CI: 1.01–1.03) were found to be risk factors of poor prognosis of AIS during follow-up (Figure 4A). Hypertension (OR: 3.71, 95% CI: 1.25–11), diabetes mellitus (OR: 4.31, 95% CI: 1.44–12.9), rtPA treatment (OR: 4.69, 95% CI: 1.25–17.6), and baseline calprotectin (OR: 1.03, 95% CI: 1.02–1.04) were found to be risk factors of disease progression of AIS during follow-up (Figure 4B).

## Discussion

In the present study, we investigated the pathological alteration and prognosis indicative effects of circulating calprotectin in AIS. We found that patients with AIS had increased plasma calprotectin levels after AIS onset, and plasma calprotectin was associated with disease severity and prognosis. Notably, no significant alteration of serum calprotectin was observed during a 2-week follow-up after AIS.

Calprotectin is a member of endogenous danger-associated molecular patterns (DAMPs) that promote in?ammation through activating toll-like receptor-4 (TLR4) and the receptor for advanced glycation end products (RAGE), which play pivotal roles in the development and progression of atherosclerosis. Calprotectin is an endogenous ligand of TLR4 and RAGE and its proatherogenic properties have been well-recognized (11). Increased calprotectin expression was found in animal and human atherosclerotic plaques, and circulating calprotectin levels are suggested to be associated with the severity of coronary artery disease and thickness of carotid intima-media (12–15). Recently, a panel of studies have discovered the association between calprotectin levels and various atherosclerosis-related diseases, including diabetes (16), unstable angina (17), and coronary heart disease (18). It has been demonstrated that calprotectin is a potential predictive factor for long-term cardiovascular events (14). Inhibition of calprotectin signaling improves cardiac function after myocardial infarction in animal models (19). These lines of evidence imply that calprotectin might play a pivotal role in the pathogenesis of cardiovascular diseases. Although previous animal studies have demonstrated that calprotectin could promote neuroinflammation and exacerbate the pathology of AIS (20), limited evidence is available in human studies, with only two studies investigated the association between calprotectin and prognosis of stroke (21, 22). Specifically, Guo et al. investigated the association between baseline calprotectin levels and 3-month functional outcomes of AIS. Marta-Enguita et al. found that plasma calprotectin is an independent predictor of 3-month mortality of AIS. These findings along with ours imply that circulating calprotectin may represent a reliable biomarker of the prognosis of AIS. Furthermore, we found in this study that the concentrations of circulating calprotectin were relatively stable during a 2-week follow-up, suggesting that the timepoint of blood sampling for determination of calprotectin is not determinant for the reliability of this biomarker. This is of significance for the variable time from symptom onset to patient admission after AIS onset.

Acute ischemic stroke shares similar pathological mechanisms with cardiac infarction, therefore we speculate that circulating calprotectin might also be associated with the severity or prognosis of AIS. As expected, we found that plasma calprotectin levels were increased in patients with AIS. Furthermore, calprotectin levels were higher in patients with poor prognosis, as indicated by mRS ≥ 3 or death, in comparison with patients with a good prognosis. Circulating calprotectin concentrations were positively correlated with the infarction volume and NIHSS score at baseline. In our regression models, baseline plasma calprotectin is predictive for short-term functional outcomes, with adjustment of possible confounding factors. The first regression model also identified calprotectin as a risk factor of poor prognosis, as indicated by mRS ≥ 3 or death. In the second regression model, we found that calprotectin is a risk factor for “disease progression,” as defined as an increase of NIHSS score during follow-up. Therefore, we could conclude that circulating calprotectin is an independent risk factor for disease progression and poor prognosis of AIS. Furthermore, circulating calprotectin also has relatively high predictive effects on short-term functional outcomes in our ROC analyses. These findings indicate that circulating calprotectin is directly associated with the severity and short-term prognosis of AIS. Our findings are in line with findings from previous studies. Guo et al. found that high plasma calprotectin levels at baseline were associated with increased risks of poor clinical outcomes at 3 months after AIS (21). It is suggested that plasma calprotectin, along with other inflammatory factors, is an independent predictor of 3-month mortality and provides complementary prognostic information to identify patients with AIS with poor outcomes after AIS (22). These findings along with ours imply that calprotectin might serve as a valid prognostic biomarker of AIS. However, the mechanism underlying this association was not clear yet, but it's not difficult to speculate that calprotectin might exacerbate the post-stroke neuronal damage by directing inflammation, as supported by several animal studies (23, 24), and suppression of calprotectin signaling could attenuate inflammation and ischemia-induced brain damage in the murine brain (25).

In conclusion, this study for the first time identified calprotectin as a short-term prognostic biomarker of AIS. However, there are several limitations of this study. First, as discussed above, previous studies have investigated the predictive value of calprotectin for 3-month functional outcome and mortality of AIS, which would reduce the novelty of the present findings. Second, we only observed the 2-week functional outcomes of stroke, thus cannot preclude that such associations could be transient rather than long-lasting. Besides, the small sample size in this study might limit its confidence.

## Data Availability Statement

The raw data supporting the conclusions of this article will be made available by the authors, without undue reservation.

## Ethics Statement

The studies involving human participants were reviewed and approved by Investigational Review Board of the Second Affiliated Hospital of Chongqing Medical University and the People's Hospital of Chongqing Hechuan (Approval No. L2017HCDH0912). The patients/participants provided their written informed consent to participate in this study.

## Author Contributions

TS, HR, and ZH designed the study and drafted the manuscript. ZH, HL, YZ, and JZ collected the samples and analyzed the data. ZH and TS supervised the project. XY, RH, and YL were responsible for the clinical assessment of subjects. All authors contributed to the article and approved the submitted version.

## Funding

This study was supported by National Nature Science Foundation of China (No. 81801711 to TS and No. 81860250 to XY).

## Conflict of Interest

The authors declare that the research was conducted in the absence of any commercial or financial relationships that could be construed as a potential conflict of interest.

## Publisher's Note

All claims expressed in this article are solely those of the authors and do not necessarily represent those of their affiliated organizations, or those of the publisher, the editors and the reviewers. Any product that may be evaluated in this article, or claim that may be made by its manufacturer, is not guaranteed or endorsed by the publisher.
